# Effects of Gouda cheese and *Allium hookeri* on thermogenesis in mice

**DOI:** 10.1002/fsn3.2115

**Published:** 2021-01-06

**Authors:** Yong‐An Kim, Sang‐Soo Lee, Jayeon Yoo, Eun‐Mi Kim, Myoung Soo Nam, Kee K. Kim

**Affiliations:** ^1^ Department of Biochemistry Chungnam National University Daejeon Korea; ^2^ National Institute of Animal Science RDA Wanju‐gun Jeolabuk‐do Korea; ^3^ Department of Predictive Toxicology Korea Institute of Toxicology Daejeon Korea; ^4^ Division of Animal Resource Science Chungnam National University Daejeon Korea

**Keywords:** *Allium hookeri*, brown adipose tissue, cheese, gastrocnemius muscle, thermogenesis, white adipose tissue

## Abstract

Cheese contains various beneficial nutrients, including calcium and whey protein, as well as large amounts of saturated fatty acids. Thus, intake of cheese increases the production of low‐density lipoprotein‐cholesterol (LDL‐C), a well‐defined risk factor for cardiovascular disease. Therefore, identification of natural products that inhibit LDL‐C production following cheese intake and verification of the efficacy of such products in animal models are essential. Here, we evaluated the effects of *Allium hookeri*, a well‐known traditional herbal remedy, on metabolism and thermogenesis in mice consuming a cheese‐containing diet. Intake of *A. hookeri* extracts significantly blocked increases in body weight and fat mass caused by intake of Gouda cheese in mice. Additionally, increases in blood triglyceride levels following intake of Gouda cheese were alleviated by *A. hookeri*. Moreover, intake of Gouda cheese enhanced thermogenesis efficiency. Thus, *A. hookeri* may have applications as an important additive for reducing the risk of metabolic disease resulting from cheese consumption.

## Graphical abstract

Identification of natural products that inhibit LDL‐C production following cheese intake and verification of the efficacy of such products in animal models are essential. Intake of *Allium hookeri* extracts significantly blocked increases in body weight and fat mass caused by intake of Gouda cheese in mice. Moreover, intake of Gouda cheese enhanced thermogenesis efficiency. Thus, *A. hookeri* may have applications as an important additive for reducing the risk of metabolic disease resulting from cheese consumption.

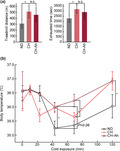

## 1 INTRODUCTION

Cheese is a rich source of calcium, protein, phosphorus, sodium, and saturated fat. In individuals who are lactose intolerant, cheese may be a good alternative to milk for maintaining bone health. However, in the last two decades, many studies have evaluated the potential effects of cheese intake on cardiovascular disease (CVD) risk factors in animal and human studies. Additionally, prospective observational studies have examined whether long‐term cheese consumption affects the development of CVDs (Chen et al., 2017; Hauswirth et al., 2005). The association between cheese consumption and disease risk has been investigated in many epidemiological studies; however, the findings have been inconsistent (Tong et al., 2017). Moreover, to improve the nutritional value of foods, food industries have started to manufacture functional foods containing bioactive compounds of plant origin (Barr & Wright, 2010; de Oliveira et al., 2020). Thus, fortification of cheese with polyphenols of plant origin may improve the quality and nutritional value of cheese and help prevent CVDs.

*Allium hookeri* contains abundant nutrients and particularly high levels of sulfur‐containing compounds, such as allicin, S‐allylcysteins, and cycloalliin. The beneficial effects of this herb are attributed to the presence of various phenols (e.g., ferulic acid, gallic acid, and cinnamic acid), phytosterols, linoleic acid, and organosulfur compounds. *Allium hookeri* is known to have bioactive properties, such as antioxidant (Cho et al., 2015; Kim et al., 2016), antimicrobial (Lucchini et al., 2018; Poznanski et al., 2004), antidiabetic (Roh et al., 2016; Yang et al., 2016), anti‐obesity (Park et al., 2018), anti‐inflammatory (Schwendimann et al., 2020), and hepatoprotective effects (Kim et al., 2019; Park et al., 2018). Recently, *A. hookeri* extract was shown to decrease body weight and improve lipid profiles in rats fed a high‐fat diet (Jang et al., 2018; Kim et al., 2019). However, no studies have investigated the effects of *A. hookeri* on the regulation of diet‐induced thermogenesis (DIT), that is, the process through which calories ingested from the food are burned off as heat rather than used to digest, absorb, and metabolize the rest of the food.

Brown adipose tissue (BAT)‐mediated metabolism functions through DIT to maintain energy homeostasis, playing pivotal roles in the regulation of thermogenesis and energy metabolism in mammals. BAT‐mediated thermogenesis is enhanced by exposure to cold conditions and high‐calorie diets, suggesting that BAT is a central effector of energy balance (Raiha et al., 2002). Moreover, BAT‐mediated thermogenesis involves crosstalk with other organs (e.g., white adipose tissue [WAT] and skeletal muscle), and this process is related to health and disease.

In this study, we investigated the effects of *A. hookeri* on thermogenesis and lipogenesis/lipolysis‐related gene expression levels in BAT, WAT, and skeletal muscle of mice consuming a cheese‐containing diet.

## 2 MATERIALS AND METHODS

### 2.1 Animals and experimental diets

Male BALB/c mice (aged 8 weeks; Daehan Biolink) were housed under controlled conditions (22 ± 2°C, relative humidity 50% ± 5%, and 12‐hr light/dark cycle), with ad libitum access to standard rodent chow and water. All mice were acclimated for 7 days before use in experiments. Eighteen mice were randomly divided into three groups (*n* = 6 mice/group): normal diet (ND), normal diet with 40% cheese (CH), and CH with 5% *A. hookeri* (200 mg/kg; CH‐Ah). During the 6‐week feeding period, food intake and body weight were recorded every 7 days. Animal care and research protocols were approved by the Animal Experimental Ethics Committee of Chungnam National University (Daejeon, Korea) and were performed in accordance with institutional guidelines.

### 2.2 Experimental diet preparation Allium hookeri extracts

*Allium hookeri* was purchased from Cheongsong, Gyeongsangbuk‐do, Korea. Dried Allium hookeri (25 g) was boiled with 1 L of distilled water for 2 hr. The water was evaporated, and the extracts were freeze‐dried. Cheeses used in this study were gouda cheese, and gouda cheese contained 5% *A. hookeri* extracts and both are fermented for 6 months.

### 2.3 Exercise treadmill test and thermogenesis test

For the exhaustive treadmill exercise test, running was initiated at a rate of 10 cm/s for 3 min with a 0% slope. The speed was then gradually increased to 15 and then 45 cm/s, and this speed was maintained until exhaustion, which was defined as the inability to run for 10 s (Yu et al., 2014). To evaluate metabolic efficiency related to diet‐induced thermogenesis, body temperature was measured in ad libitum‐fed mice after cold exposure at 4°C for 120 min (*n* = 6/group).

### 2.4 Serum biochemical analysis

The effects of cheese with *A. hookeri* supplementation on serum triglyceride, total cholesterol, high‐density lipoprotein‐cholesterol (HDL‐C), low‐density lipoprotein‐cholesterol (LDL‐C), glutamic‐oxaloacetic transaminase (GOT), glucose, blood urea nitrogen (BUN), and creatine kinase levels were determined using an autoanalyzer (TBA‐40FR; Toshiba). Tukey's tests were used to assess significance, and results with *p* values less than .05 were considered significant.

### 2.5 Gene expression analysis

Total RNA was isolated from untreated and treated gastrocnemius muscles (GAS) and cells using GeneAll Hybrid‐R RNA Purification Kit (GENEALL). After RNA isolation, complementary DNA (cDNA) was synthesized using a M‐MLV reverse transcriptase (Promega) using random hexamers. Accumulated polymerase chain reaction (PCR) products were detected by monitoring increases in SYBR reporter dye fluorescence. The PCR primers used in this study were 5′‐GGCCCTTGTAAACAACAAAATAC‐3′ and 5′‐GGCAACAAGAGCTGACAGTAAAT‐3′ for *Ucp1* and 5′‐ACCATGACTACTGTCAGTCACTC‐3′ and 5′‐GTCACAGGAGGCATCTTTGAAG‐3′ for *Pgc1* and 5′‐CCACCAGCGAGGACTTCAC‐3′ and 5′‐GGAGGACTCTCGTAGCTCGAA‐3′ for *PRDM16* and 5′‐AGTCCCTGAATGATAACACG‐3′ and 5′‐AAGCACCTTCCGCAATAT‐3′ for *Cidea* and 5′‐GGAGGTGGTGATAGCCGGTAT‐3′ and 5′‐TGGGTAATCCATAGAGCCCAG‐3′ for *Lpl* and 5′‐CAAGAACAGCAACGAGTACCG‐3′ and 5′‐GTCACTGGTCAACTCCAGCAC‐3′ for *C/ebp* and 5′‐GGGAGTTTGGCTCCAGAGTTT‐3′ and 5′‐TGTGTCTTCAGGGGTCCTTAG‐3′ for *Fas* mRNA. Quantitative RT‐PCR (qRT‐PCR) was performed in AriaMX Real‐time PCR System (Agilent Technologies) using the following conditions: initial denaturation at 95°C for 3 min, followed by 40 cycles of 95°C for 20 s, 58°C for 20 s, and 72°C for 20 s. The specificity of PCR products was confirmed by melting curve analysis of qRT‐PCR and agarose gel electrophoresis of the PCR product.

### 2.6 Statistical analysis

All data are expressed as means ± standard errors of the means of at least three separate experiments for each group. Comparisons between experimental groups were made using one‐way analysis of variance, following Turkey's multiple range test (SPSS 12.0 software; BMI). Mean values were considered significantly different when *p* values were less than .05.

## 3 RESULTS

### 3.1 Physical characteristics and experimental diets

**FIGURE 1:**
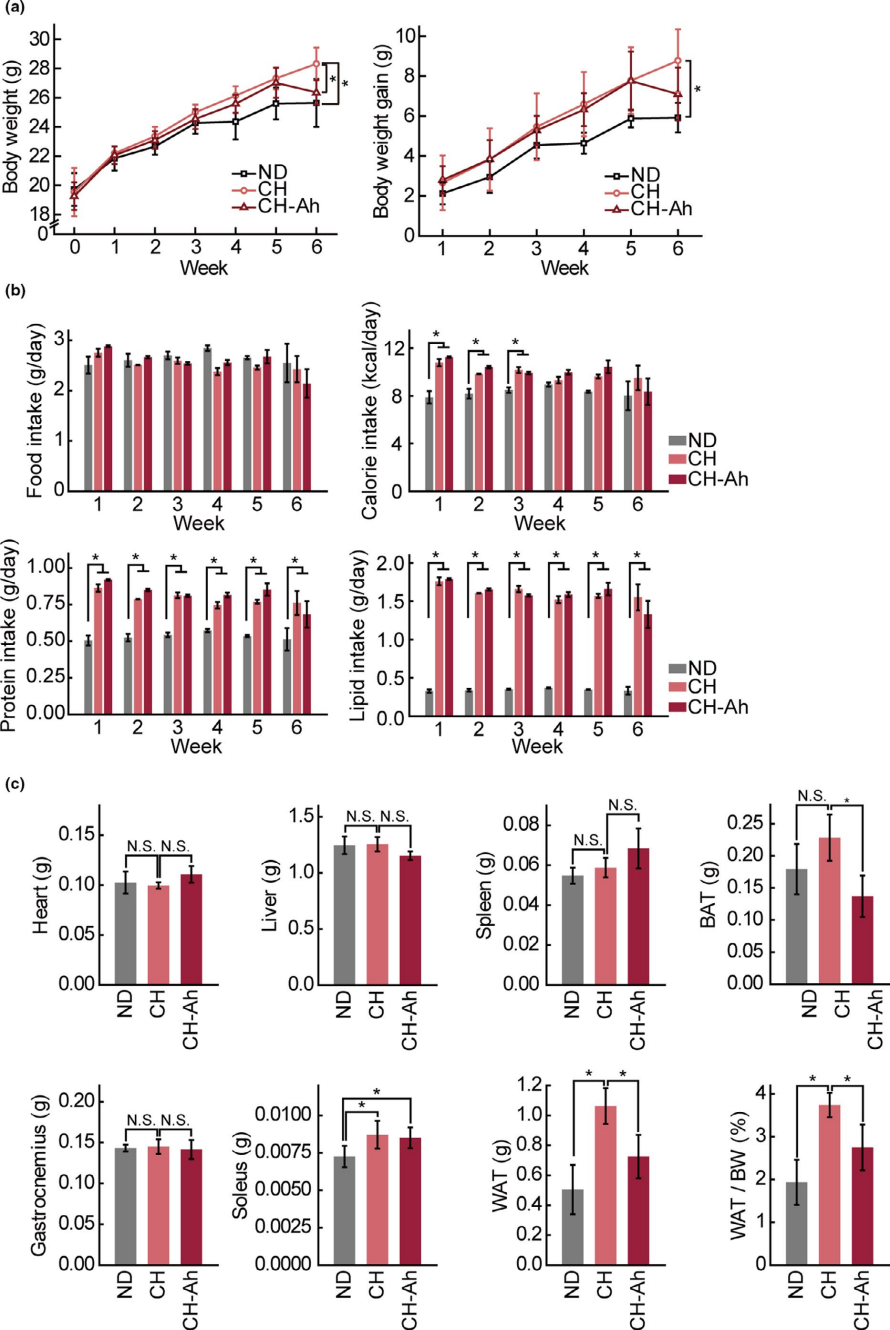
Changes in body weight gain, food intake, food efficiency ratio, and relative organ weights of mice fed experimental diets. Mice   were provided a normal diet, cheese (40% of feed), or cheese‐containing *Allium hookeri* (40% of feed) for 6 weeks. All experimental data are presented as means ± standard deviations (n = 6/group, NS, no statistical significance; *, *P* < .05; paired t‐test)

To investigate whether cheese with *A. hookeri* improved thermogenesis, we first measured body weight, organ mass, and food intake to determine the phenotype of metabolic efficiency. CH‐fed mice showed higher body weights and weight gain than the ND‐fed group (Figure 1a). Food intake did not differ among the groups. In addition, the calorie intake of the CH‐fed group was higher than that of the ND‐fed group (Figure 1b). Notably, although CH‐Ah‐fed mice had higher calorie intake, their body weight and weight gain were significantly lower than those of CH‐fed mice. And then parallel with body weight, WAT mass of CH‐Ah fed group had significantly lower compared with CH‐fed group. Moreover, as shown in Figure 1c, CH‐ and CH‐Ah‐fed groups had significantly higher soleus muscle mass than ND‐fed mice. However, the weights of other organs were not affected by CH or CH‐Ah (Figure 1c).

### 3.2 Serum levels of lipids, creatine kinase, GOT, BUN, and glucose

**FIGURE 2:**
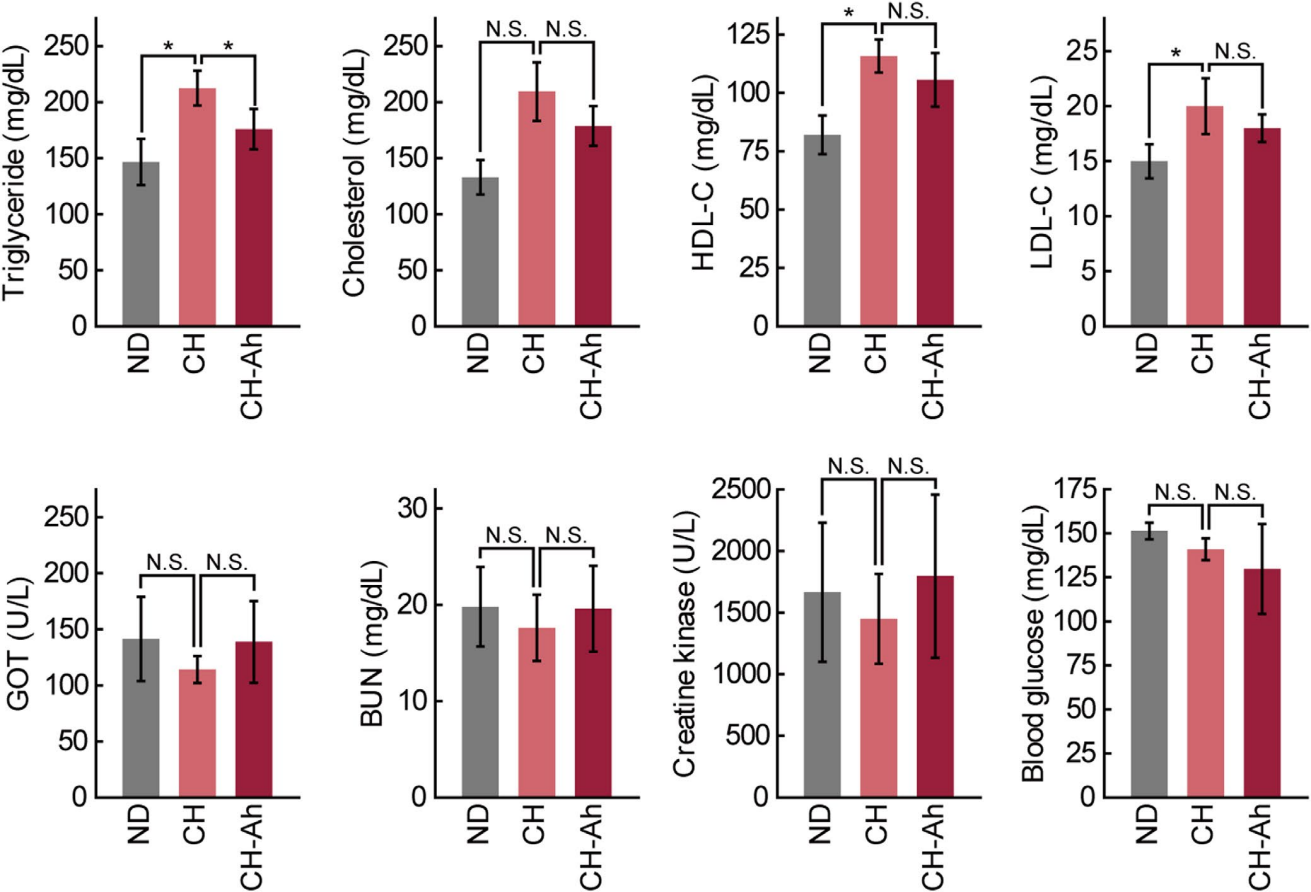
Serum lipid, creatine kinase, GOT, BUN, glucose, HDL, and LDL levels in mice fed experimental diets. BUN, blood urea nitrogen; GOT, glutamic‐oxaloacetic transaminase; HDL, high‐density lipoprotein; LDL, low‐density lipoprotein (*n* = 6/group, NS, no statistical significance; *, *P* < .05; paired t‐test)

Figure 2 shows the serum lipid profiles of the mice fed CH or CH‐Ah. CH feeding significantly increased triacylglycerol levels compared with ND feeding; however, the triglyceride levels in the CH‐Ah group were significantly decreased by 160 ± 5.7 mg/dl compared with those in the CH group. The levels of total cholesterol, HDL‐C, and LDL‐C were significantly increased in the CH group compared with the ND group. However, there were no significant differences in the levels of total cholesterol, HDL‐C, and LDL‐C between the CH and CH‐Ah groups. GOT, BUN, creatine kinase, and glucose levels did not differ among groups (Figure 2).

### 3.3 Exercise function and cold exposure tests

**FIGURE 3:**
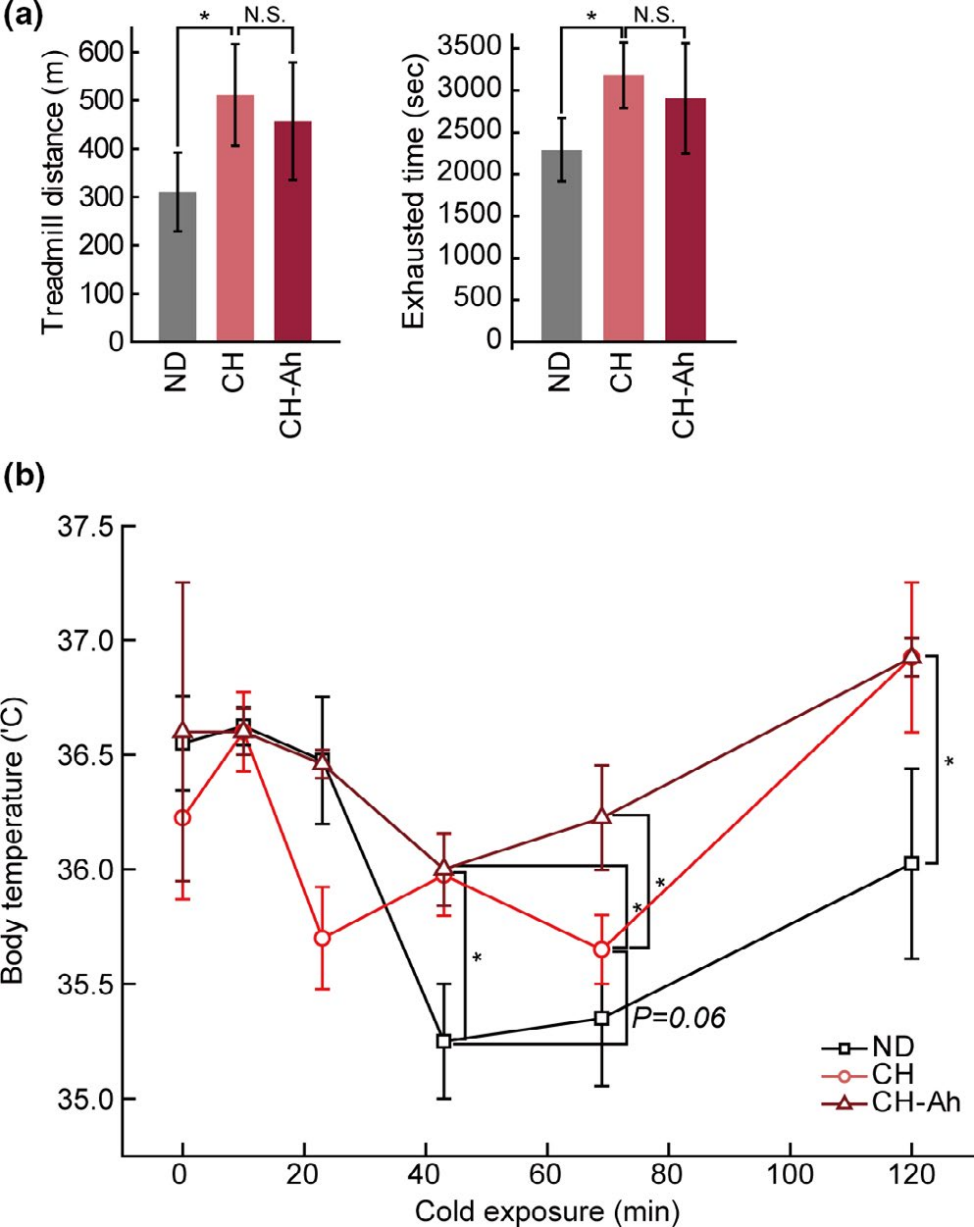
Effects of cheese with *Allium hookeri* on exercise function and thermogenesis. Metabolic effects of cheese and cheese with *A. hookeri* in (a) endurance exercise tests and (b) core temperature analyses. Data are presented as means ± standard errors (*n* = 6/group, NS, no statistical significance; *, *P* < .05; paired t‐test)

Next, we evaluated the effects of CH with *A. hookeri* supplementation on exercise function. Mice in the CH and CH‐Ah groups showed no significant differences in time to exhaustion or distance travelled compared with mice in the ND group (Figure 3a). To further elucidate the effects of CH with *A. hookeri* on metabolic efficiency in mice, we measured core temperatures in mice after cold exposure (4°C) for 2 hr. The results showed that all groups had lower body temperatures after 60 min, although that in mice in the CH‐Ah group was significantly higher than the body temperatures of mice in the ND and CH groups (Figure 3b).

### 3.4 mRNA levels of metabolism‐related genes in the BAT, WAT, and GAS

**FIGURE 4:**
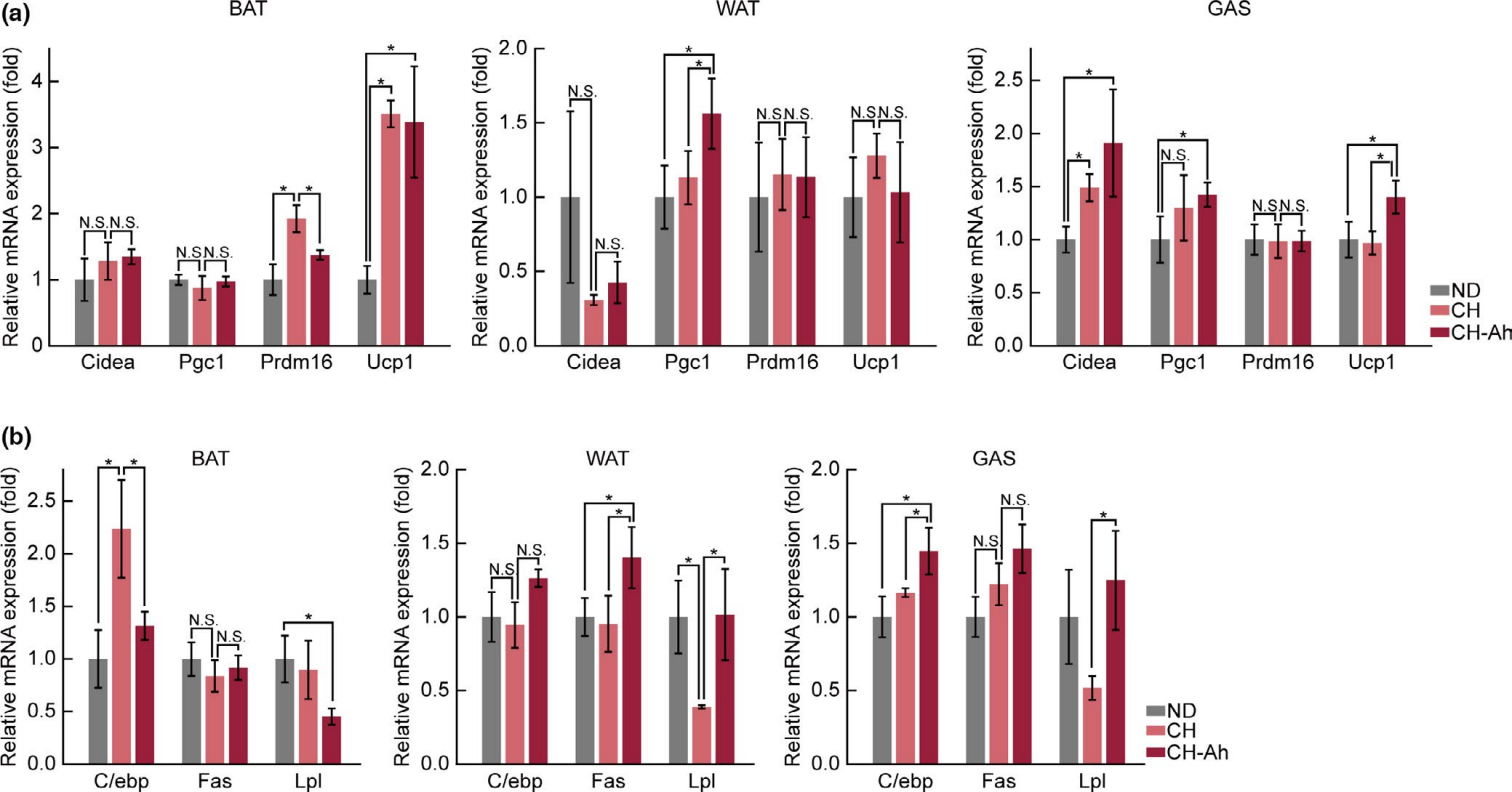
Effect of cheese with *Allium hookeri* on mRNA levels of metabolism‐related genes in BAT, WAT, and GAS. FAS, fatty acid synthase; LPL, lipoprotein lipase; C/EBPα, CCAAT/enhancer‐binding protein α(*n* = 6/group, NS, no statistical significance; *, *P* < .05; paired t‐test)

A major function of BAT is dissipation of energy as heat. To investigate the mechanisms through which *A. hookeri* facilitates the retention of core temperature under cold conditions, we assessed the expression levels of thermogenesis‐associated genes by quantitative PCR. Notably, our results showed that the levels of uncoupling protein 1 (*Ucp1*) mRNA were significantly increased in the BAT, WAT, and GAS of mice in the CH and CH‐Ah groups compared with those in the ND group. As a marker of mitochondrial biogenesis, peroxisome proliferator‐activated receptor γ coactivator 1 (*Pgc1*) mRNA levels were also significantly increased in parallel with *Ucp1* levels in the WAT and GAS (Figure 4a).

.We also investigated the mechanisms through which *A. hookeri* reduced body weight, weight gain, WAT mass, and serum triacylglycerol levels to determine whether lipolysis was promoted in the WAT and GAS in the CH‐Ah group. As a marker of lipolysis, the levels of lipoprotein lipase (*Lpl*) mRNA were significantly decreased in the CH group compared with those in the ND group and significantly increased in the CH‐Ah group compared with those in the CH group (Figure 4b).

## 4 DISCUSSION

Cheese is a dairy product commonly consumed worldwide. However, adjustment of the amount of cheese intake may be required to prevent CVDs owing to the excess fat contained in cheese. Changes in weight and fat indices reflect the status of body metabolism after diet meal supplementation in experimental models. Metabolic energy expenditure can be divided into three processes: basal metabolic rate (BMR), DIT, and active metabolic rate (AMR) (Barr & Wright, 2010). BMR is the energy cost associated with maintaining body function at rest, and AMR is the energy expenditure resulting from daily physical activities. DIT is the body's increase in metabolism following the ingestion of food (Trexler et al., 2014). Importantly, DIT accounts for the energetic costs of postprandial processes, such as food breakdown, enzyme synthesis, peristalsis, nutrient uptake/assimilation, and secondary metabolism (e.g., urea synthesis), and is typically responsible for approximately 10% of daily energy expenditure in humans (Muller et al., 2016).

Brown adipose tissue is a major site of nonshivering thermogenesis and is critical for the maintenance of body temperature (Chouchani et al., 2019; Foster & Frydman, 1978). BAT‐mediated nonshivering thermogenesis is increased by exposure to high‐calorie diets (diet‐induced thermogenesis), suggesting that BAT is a central effector of energy balance (Bachman et al., 2002). Useful markers of BAT include UCP1, PGC1α, cell death‐inducing DFFA‐like effector A (CIDEA), and PRD1‐BF1‐RIZ1 homologous domain containing 16 (PRDM16) (Xi et al., 2019). Mitochondrial UCP1 acts as a valve to rapidly release this energy as heat instead of synthesizing ATP. Intracellular levels of purine nucleoside diphosphates and triphosphates constitutively inhibit UCP1, whereas increasing intracellular‐free fatty acid levels induced by cold stimulation acutely activate UCP1 (George & Heaton, 1978). PGC1α regulates thermogenesis by directly inducing the expression of UCP1. UCP1 and PGC1α activate a number of other transcription factors and function as central regulators of numerous pathways involved in mitochondrial biogenesis and thermogenesis (Finck & Kelly, 2006). PRDM16 controls a bidirectional cell fate switch between skeletal myoblasts and brown fat cells. Loss of PRDM16 from brown fat precursors causes loss of brown fat characteristics and promotes muscle differentiation. PRDM16‐deficient brown fat exhibits an abnormal morphology, reduced thermogenic gene expression, and elevated expression of muscle‐specific genes (Seale et al., 2008). Manipulation of fat storage is an obvious therapeutic objective; however, disruption of the normal differentiation or development of WAT causes ectopic lipid storage and severe pathology (lipodystrophy) in cheese supplemented with *A. hookeri*. CIDEA is a member of the CIDE apoptotic family and is highly expressed in BAT. Moreover, CIDEA directly interacts with UCP1 and suppresses its uncoupling function, thereby reducing energy expenditure. CIDEA‐deficient mice are resistant to obesity and show higher metabolic rates in BAT. Additionally, CIDEA‐null mice also have higher body temperatures when exposed to cold compared with wild‐type mice (Zhou et al., 2003), suggesting that thermogenesis is increased in the absence of CIDEA. CIDEA has been shown to regulate the metabolic sensor AMP‐activated protein kinase (AMPK), which plays critical roles in energy homeostasis. CIDEA forms a complex with the β subunit of AMPK, which leads to ubiquitin‐mediated degradation of the AMPK‐β subunit. In the absence of CIDEA, the protein levels and enzymatic activity of AMPK are increased in BAT (Qi et al., 2008). Therefore, CIDEA not only regulates UCP1 activity but also controls AMPK‐associated energy homeostasis.

In this study, UCP1, a marker of nonshivering thermogenesis, showed significant upregulation in the CH‐Ah group compared with that in the ND and CH groups in BAT and GAS. In addition, PGC1, a marker of mitochondrial biogenesis, was also significantly upregulated in the CH‐Ah group compared with that in the ND and CH groups in WAT. Therefore, these results suggested that maintenance of core temperature by cold exposure was involved in modulation of thermogenesis homeostasis.

CCAAT/enhancer‐binding protein (C/EBP)α mRNA and its protein products C/EBPα are expressed in BAT (Edwards et al., 2000) and play important roles in regulating pre‐adipocyte differentiation and promoting the expression of lipogenesis‐related genes (Kaur et al., 2007). Fatty acid synthase (FAS) catalyzes the de novo synthesis of fatty acids. FAS, storage, and metabolism are essential during thermogenesis because they are required for UCP1 protein transport activity in BAT (Bartelt et al., 2012; Fedorenko et al., 2012). The lipoprotein lipase (LPL) is a bioactive lipid generated by the phospholipase A family of lipases, which is believed to have important roles in several diseases (Pineiro & Falasca, 2012). In this study, C/EBP and LPL levels were significantly decreased in the CH‐Ah group compared with the ND and CH groups in BAT. However, C/EBP and LPL levels in the GAS were significantly higher in the CH‐Ah group than in the ND group. In addition, FAS and LPL levels were significantly increased in the CH‐Ah group compared with the ND and CH groups in WAT. Therefore, these results suggested that loss of fat mass following *A. hookeri* supplementation was involved in modulation of lipogenesis and lipolysis.

The specific peripheral metabolic organs involved in maintenance of metabolism fitness include the liver, muscle, WAT, BAT, and pancreas (Castillo‐Armengol et al., 2019). In this study, we focused on organismal adaptation to the consumption of food, followed by the thermogenesis response engaged in high‐energy conditions. Our results showed that cheese prepared using *A. hookeri* improved lipid profiles in the serum and decreased body weight gain and fat weight. Moreover, *A. hookeri* supplementation inhibited the formation of triacylglycerol in the serum, increased the maintenance of body temperature in a cold environment, and increased the activity of thermogenesis‐related genes in BAT, WAT, and GAS. Overall, the lipid metabolism‐improving properties of *A. hookeri* are thought to be related to the inhibition of obesity and CVDs.

In summary, the inclusion of *A. hookeri* in cheese may have benefits over commercial cheese with respect to anti‐obesity effects and improvement of lipid metabolism. However, further studies are required to clarify the detailed mechanisms underlying the anti‐obesity effects of cheese supplemented with bioactive compounds, including *A. hookeri*.

## Data Availability

The data that support the findings of this study are available from the corresponding author upon reasonable request.
